# EMG space similarity feedback promotes learning of expert-like muscle activation patterns in a complex motor skill

**DOI:** 10.3389/fnhum.2022.805867

**Published:** 2023-01-20

**Authors:** Victor R. Barradas, Woorim Cho, Yasuharu Koike

**Affiliations:** ^1^Institute of Innovative Research, Tokyo Institute of Technology, Yokohama, Japan; ^2^School of Engineering, Tokyo Institute of Technology, Yokohama, Japan

**Keywords:** motor learning, biofeedback, muscle synergies, complex motor skills, virtual reality

## Abstract

Augmented feedback provided by a coach or augmented reality system can facilitate the acquisition of a motor skill. Verbal instructions and visual aids can be effective in providing feedback about the kinematics of the desired movements. However, many skills require mastering not only kinematic, but also complex kinetic patterns, for which feedback is harder to convey. Here, we propose the electromyography (EMG) space similarity feedback, which may indirectly convey kinematic and kinetic feedback by comparing the muscle activations of the learner and an expert in the task. The EMG space similarity feedback is a score that reflects how well a set of muscle synergies extracted from the expert can reconstruct the learner’s EMG when performing the task. We tested the EMG space similarity feedback in a virtual bimanual polishing task that uses a robotic system to simulate the dynamics of a real polishing operation. We measured the expert’s and learner’s EMG from eight muscles in each arm during the real and virtual polishing tasks, respectively. The goal of the virtual task was to smoothen the surface of a virtual object. Therefore, we defined performance in the task as the smoothness of the object at the end of a trial. We separated learners into real feedback and null feedback groups to assess the effects of the EMG space similarity feedback. The real and null feedback groups received veridic and no EMG space similarity feedback, respectively. Subjects participated in five training sessions on different days, and we evaluated their performance on each day. Subjects in both groups were able to increase smoothness throughout the training sessions, with no significant differences between groups. However, subjects in the real feedback group were able to improve in the EMG space similarity score to a significantly greater extent than the null feedback group. Additionally, subjects in the real feedback group produced muscle activations that became increasingly consistent with an important muscle synergy found in the expert. Our results indicate that the EMG space similarity feedback promotes acquiring expert-like muscle activation patterns, suggesting that it may assist in the acquisition of complex motor skills.

## 1. Introduction

Most complex motor skills involve interacting with objects in the environment. These motor skills require generating not only precise movements, but also precise forces. In fact, inappropriate forces may lead to failure in the task. For instance, an experienced nylon-string guitar player may face difficulties playing a familiar piece on a steel-string guitar due to the higher stiffness of steel strings. In this case, even though the required hand movements are kinematically equivalent, the kinetic (force) requirements differ considerably.

Therefore, to master such complex motor skills, the kinematic and kinetic components of the task must be learned concurrently. Appropriate sensory feedback about the execution of the task is essential for effective training (Kernodle and Carlton, 1992; Young and Schmidt, 1992; Zubiaur et al., 1999; Hinder et al., 2008). In particular, augmented feedback from a coach (Reid and Giblin, 2015) or an augmented reality system (Emken and Reinkensmeyer, 2004, 2005; Sigrist et al., 2013) may assist the learner in evaluating performance aspects that are not easy to self-evaluate (Lauber and Keller, 2014). Providing augmented kinematic feedback about task execution is relatively simple. A common coaching technique is to guide and/or evaluate motion trajectories that the learner generates. These evaluations may be verbal or visual, relying on in-person demonstrations, or on video recordings of an expert performer or of the learners themselves (Al-abood et al., 2001). In contrast, providing augmented kinetic feedback is in general a more complex problem, requiring especially instrumented equipment to measure forces or torques (Broker et al., 1993; Smith and Loschner, 2002). Without such equipment, augmented kinetic feedback is limited to verbal instructions about the desired forces in the task. However, verbal instructions may not be as effective in promoting learning of kinetic tasks as feedback derived from instrumented equipment (Dowling et al., 2010).

Here, we explore a new kind of augmented feedback based on electromyography (EMG) measurements. EMG from muscles involved in a task can be used to estimate the stiffness of their corresponding limb (Osu et al., 2002; Shin et al., 2009). According to the theory of impedance control, the CNS can concurrently satisfy kinematic and kinetic task goals by adjusting muscle activations to modulate the stiffness of the end-point of the limb (Hogan, 1985b). This allows the CNS to control the compliance of the limb to external forces and achieve successful interactions with the environment (Hogan, 1985a; Burdet et al., 2001). Muscles are also responsible for generating movement, and consequently EMG signals contain information about both the kinetics and the kinematics of movements in a task.

Therefore, we hypothesize that augmented feedback that quantifies the similarity of the muscle activations of a learner and the muscle activations of an expert in a task can enhance skill learning by promoting the acquisition of expert-like muscle activation patterns. This in turn may promote learning of the kinetic components of the task. We used muscle synergy analysis (D’Avella et al., 2003) to identify the muscle activation patterns of the expert, and used these patterns to reconstruct the learner’s muscle activations. We defined the resulting reconstruction quality as the EMG space similarity, which we provided to the learner as a score during training in the task. Higher scores indicate that the muscle activations of the learner and the expert reside in spaces that overlap, which may be associated with the acquisition of expert-like muscle activation patterns.

We tested our hypothesis in the context of a simulated polishing operation. Expertise in polishing operations entails maintaining the attack angle between the grinder and the polished object as constant as possible (Tsujiai et al., 2015). This requires the generation of precise force patterns that counteract the interaction forces that arise when pressing the polished object against the grinder (Kodama et al., 2015). To test our hypothesis, we designed a virtual polishing task that simulates the dynamic environment of a polishing operation and provides the EMG space similarity score to learners in real time. The EMG space similarity score was computed based on the muscle activation patterns of a polishing expert.

## 2. Materials and methods

### 2.1. Subjects

Twenty-one subjects [20 learners: mean age, 28.2 yr (SD 8.9), 19 males, 19 right-handed (self-reported); 1 expert: 59 yr, male, right-handed] participated in the study after providing written informed consent. All procedures were approved by the Ethical Review Board of the Tokyo Institute of Technology.

### 2.2. Experimental setup

**FIGURE 1:**
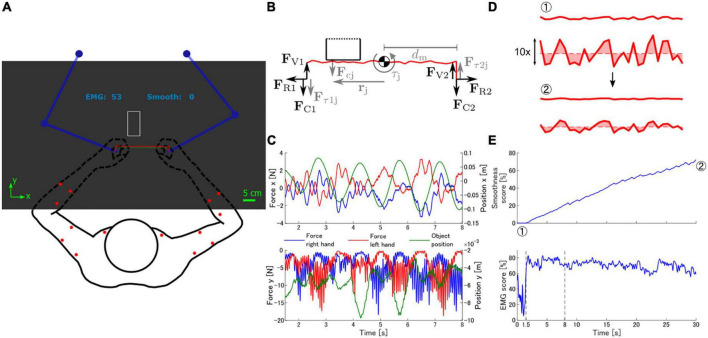
Virtual task environment. **(A)** Experimental setup. Participants held a pair of manipulanda (blue), and moved them to control the position of the polished object (red line). The main goal of the task was to smoothen the polished object by moving it against the grinder (white rectangle). Contact between both objects caused the manipulanda to generate forces simulating the contact interaction, which also changed the shape of the polished object. The EMG and smoothness scores were displayed above the task workspace. Red dots on the arms indicate the location of the EMG electrodes. **(B)** Simulation of polishing task dynamics. The robotic manipulanda applied forces on the participants’ arms according to three components: forces to keep the distance between hands constant simulating a rigid object (F_Ri_), contact between the polished object and the grinder (F_Ci_), and vibration forces (F_Vi_). Contact forces F_cj_ originated from the overlap of the vertices of the polished object and the grinder and were applied to both arms. Contact forces also generated torque (**τ**_j_) around the centroid of the polished object according to the distance between the contact point and the centroid (r_j_). The torque **τ**_j_ was simulated by a pair of forces applied to both arms (F_**τ**ij_). Black arrows represent the main force components (F_R_, F_C_, F_V_). Gray arrows represent sub-components of the main forces. **(C)** Forces generated by the robotic manipulanda and position of the virtual object during a sample portion of a trial in the virtual polishing task. Notice that the magnitude of the vertical forces applied on each hand is coupled to the movement of the object along the horizontal axis. The position of the object is measured with respect to the center of the front edge of the grinder. **(D)** Deformation of the polished object by contact forces with the grinder. The polished object is shown with and without scaling in the vertical direction. The smoothness of the object was quantified as the area between the outline of the object and a horizontal line at the mean vertical position of all contact points. **(E)** Smoothness and EMG similarity scores throughout a trial. The objects in panel **(D)** correspond to the points indicated by circled numbers. Vertical dashed lines indicate the portion of the trial displayed in panel **(C)**.

We designed a bimanual virtual task that simulates a polishing operation with a bench grinder. Participants sat facing a pair of planar robotic manipulanda (KINARM End-point Lab; BKIN Technologies) and held onto the handle of each manipulandum (Figure 1A). A virtual environment was displayed on a mirror placed on a horizontal plane above the participant’s arms, occluding vision of the arms and the manipulanda. The mirror reflected images displayed on a screen placed above it. The virtual environment consisted of a polished object (object with irregular shape), a grinder (rectangular object), and score displays (EMG and smoothness scores or smoothness score only). Participants were able to control the position of the polished object by moving the manipulanda. Arm end-point movements were constrained to a horizontal plane at approximately the height of the xiphoid process of the sternum.

We recorded surface EMG activity from eight muscles in each arm: deltoid posterior (DeltP), pectoralis major (PectMaj), triceps brachii long head (TriLong), biceps brachii long head (BicLong), brachioradialis (BrRad), palmaris longus (PalmLong), extensor digitorum (ExtDig) and extensor carpi radialis (ExtCR). Active bipolar electrodes (Trigno, analog output mode; Delsys) were used to record EMG activity. EMG signals were bandpass filtered (20–450 Hz) *via* hardware, and digitized at 2 kHz using an analog-to-digital converter coupled to the KINARM’s real time computer. Further EMG processing included rectification, low-pass filtering (second order Butterworth filter, 5 Hz) and normalization. EMG signals were normalized to the maximum EMG activity of each muscle during a maximum voluntary contraction (MVC) task, as described in the section “2.7. Experimental protocol.”

### 2.3. Virtual environment

Participants experienced a dynamic virtual environment that simulated a polishing task (Figure 1A). The polished object was represented as a jagged red line formed by connecting a set of randomly generated contact points. Thirty contact points were placed at uniform intervals along a 22 cm long straight line with a random perpendicular distance to the straight line taken from a uniform distribution (range: [−2.5, 2.5] mm). The grinder was represented as a 5 × 10 cm rectangular white outline fixed at the center of the display.

The robotic manipulanda generated forces onto the participant’s hands to simulate the interaction between objects in the polishing task (Figure 1B). The generated forces were the sum of three components:


(1) $${\text{F}}_{\text{Ti}}={\text{F}}_{\text{Ri}}+{\text{F}}_{\text{Ci}}+{\text{F}}_{\text{Vi}}$$

where **F**_T_ is the total force produced by the manipulandum, **F**_R_ is a force that simulates the rigidity of the polished object, **F**_C_ is a force arising from the contact forces between the polished object and the grinder, and **F**_V_ is a vibration force. The subindex *i* = 1, 2 indicates forces corresponding to the left and right manipulanda, respectively.

In order to approximate the sensation of holding a rigid body between both hands, the rigidity of the polished object was simulated as a pair of rigid spring-dampers. For each side, the spring-damper was attached between the end-point of the manipulandum and the end-point of the polished object:


(2) $${\text{F}}_{\text{Ri}}={\text{F}}_{\text{si}}+{\text{F}}_{\text{di}}$$

(3) $${\text{F}}_{\text{si}}=k\,\left({\text{p}}_{\text{hi}}-{\text{p}}_{\text{oi}}\right)$$

(4) $${\text{F}}_{\text{di}}=b\,\left({\text{v}}_{\text{hi}}-{\text{v}}_{\text{oi}}\right)$$

where **F**_s_ and **F**_d_ are the spring and damper forces, **p**_h_ and **p**_o_ are the positions of the manipulandum end-point and the polished object end-point, **v**_h_ and **v**_o_ are the velocities of the manipulandum end-point and the polished object end-point, and k and b are the stiffness and damping coefficients (*k* = 1000 N/m, *b* = 5 Ns/m). Therefore, the spring-damper produces equal and opposite forces on each hand that attempt to maintain the position of the manipulanda end-points close to the ends of the polished object (Figure 1C, horizontal forces).

Contact forces between the polished object and the grinder were computed based on the overlap between the polished object and the grinder. The overlap was detected when one or more contact points in the polished object entered the area defined by the outline of the grinder. Contact forces **F**_c_ were modeled as elastic forces proportional to the distance *d*_j_ between the front edge of the grinder and the contact point j:


(5) $${\text{F}}_{\text{cj}}=k\,d_{\text{j}}\,\text{y}$$

where *k* is the stiffness coefficient (*k* = 1000 N/m) and **y** is a unit vector along the y axis, indicating that contact forces were always perpendicular to the front edge of the grinder. Each of the contact forces **F**_cj_ also generated a torque **τ**_j_ around the mid-point of the polished object. The torques were computed as the cross-product of the contact force and **r**_j_, the position vector of contact point j with respect to the mid-point of the polished object.


(6) $$\tau_{\text{j}}={\text{r}}_{\text{j}}\times {\text{F}}_{\text{cj}}$$

The torques **τ**_*j*_ were simulated by converting them to a pair of opposing forces perpendicular to the polished object applied at both manipulanda end-points,


(7) $${\text{F}}_{\tau \,\text{ij}}=\frac{||\tau_{\text{j}}||}{d_{\text{m}}}\,{\text{u}}_{\text{i}}$$

where *d*_m_ is the distance between the midpoint and the end-point of the polished object, and **u** is a unit vector perpendicular to the polished object. Brackets indicate the magnitude of **τ**_j_. The directions of **u**_i_ were determined according to the direction of **τ**. The contact-related force generated by each manipulanda was defined as the sum of contact forces from all overlapping contact points and their associated torque forces:


(8) $${\text{F}}_{\text{Ci}}=\sum_{\text{j}}\left({\text{F}}_{\text{cj}}+{\text{F}}_{\tau \,\text{ij}}\right)$$

Vibration forces were produced when the polished object contacted the grinder. The amplitude of the vibration on each manipulandum was proportional to the magnitude of the total contact forces on the manipulandum (gain, *g* = 0.15) and had a frequency *f* of 15 Hz. The direction of the vibration forces was perpendicular to the grinder:


(9) $${\text{F}}_{\text{Vi}}=\text{y}\,||{\text{F}}_{\text{Ci}}||\,g\,\text{sin}\,\left(2\,\pi \,f\,t\right)$$

where t is time. Forces along the vertical axis are mainly the sum of **F**_Ci_ and **F**_Vi_ (Figure 1C, vertical forces).

The polishing effect between the polished object and the grinder was simulated by deforming the polished object according to the computed contact forces on each contact point. Each contact point j was modeled as a two-dimensional point mass-damper system (*m* = 0.1 kg, *b* = 300 Ns/m^2^) with the contact force **F**_cj_ as input (Equation 5), producing acceleration of the contact point. We used the Euler method to integrate the computed acceleration and obtain the new position of each contact point at each simulation step.

### 2.4. Smoothness score

The main goal of the task was to use the grinder to transform the initially jagged polished object into a smooth and straight line. We provided learners with feedback about the smoothness of the polished object (aside from the shape of the object itself) in the form of a smoothness score during the task. We quantified smoothness as the area between the line defined by the polished object and a straight line crossing the polished object from end to end at the mean vertical position of the contact points (Figures 1D, E). We showed the smoothness score to participants as a number between 0 and 100 in a linear scale, with 0 indicating the smoothness at the start of a trial (before polishing), and 100 indicating a perfectly smooth and flat object (area between object line and straight line is zero).

### 2.5. EMG space similarity score

An additional objective during the polishing task was to maximize the EMG space similarity score (EMG score). The EMG score reflected the similarity between the spaces containing the muscle activations of the learners and of an expert in the polishing task. This score was defined as the quality of the reconstruction of the participant’s EMG activity based on a set of muscle synergies extracted from the expert (“see the section 2.6. Expert muscle synergies”). Higher qualities of reconstruction indicate that participants generate muscle activations that could be generated by the expert during the polishing task with a high degree of plausibility, and vice versa. To calculate the quality of reconstruction we first solved a non-negative linear regression problem to find synergy activation coefficients **c** that approximate the measured muscle activations **m** based on the expert’s muscle synergies **S**


(10) $$\min_{c}{||{\text{m}}_{r}-\text{m}||}^{2}\,s.t.{\text{c}}_{i}\geq 0$$

$${\text{m}}_{r}=\text{Sc}$$

where **m**_r_ are the reconstructed muscle activations. Only synergies **S** extracted using the bimanual analysis described in the section “2.8.1. Synergy extraction” were used. We used the lsqnonneg function in Matlab to solve this non-negative linear regression problem. We then defined the quality of reconstruction as


(11) $$R^{2}=100\,\left(1-\frac{\sum_{j}\sum_{i}{\left(m_{i\,j}-m_{r\,i\,j}\right)}^{2}}{\sum_{j}\sum_{i}{\left(m_{i\,j}-{\bar{m}}_{i}\right)}^{2}}\right)$$

where m_ij_ is the activation of the i*^th^* muscle in the j*^th^* sample, m_r_ are the reconstructed muscle activations, and ${\bar{m}}_{i}$ is the mean activation of the i*^th^* muscle within a defined moving window. For a given time, the moving window contained the most recent 100 samples of **m** and their corresponding **c**, which amounts to 50 ms of data. The reconstruction quality calculated from the window was stored in an additional window that held the results of the 200 most recently computed reconstruction quality values, containing information about the latest 150 ms of data. The average value of the reconstruction quality window was shown to the participants as feedback. The sizes of the windows were selected so that participants could perceive a causal relationship between muscle contractions and the EMG score. Figure 1E shows an example of the EMG space similarity score for one trial.

### 2.6. Expert muscle synergies

We used the muscle synergies extracted from an expert as a template of the muscle synergies that participants should learn to use during a polishing task. The expert has 35 years of experience in polishing operations, and has received the title of master from an industry regulatory organization. The expert continuously engages in training apprentices. The synergies were extracted from the expert performing a polishing operation on metal cylinders using an industrial bench grinder. The real polishing environment differs from the virtual environment in that motion and forces in the virtual environment are not constrained to a horizontal plane, and in the postures of the expert and participants during the task. In the real environment, the grinder is placed approximately at knee height, allowing the expert to support their forearms on their upper legs while performing the task. However, both environments require oscillatory movements of the arms to the sides to accomplish the task. The expert performed the polishing operation in 7 trials, each lasting 60 s. EMG signals were measured and processed using the same methods described in the sections “2.2. Experimental setup” and “2.8.1. Synergy extraction.” We pooled the EMG data of all 7 trials for analysis.

### 2.7. Experimental protocol

Participants attended five experimental sessions of the virtual polishing task on different days. Sessions were scheduled according to participant availability. The mean time between sessions was 3.8 days (SD 3.0). The minimum and maximum times between sessions were 1 and 14 days, respectively. Participants completed the five sessions in 15.2 days (SD 9.3) on average. The minimum and maximum times of completion were 5 and 35 days, respectively.

Participants were divided into two groups: the real feedback and the null feedback groups (10 participants in each group). The real feedback group received veridic smoothness and EMG scores as described in the previous sections. The null feedback group received the veridic smoothness score, but did not receive the EMG score.

Each experimental session consisted of a maximum voluntary contraction task and the virtual polishing task. The MVC task was conducted before the virtual polishing task for EMG normalization purposes. In the MVC task, participants performed maximum voluntary contractions of their arm muscles by producing maximum isometric torques at the degrees of freedom of joints that are relevant to the motion in the task and the muscles used for EMG measurement (wrist flexion/extension and ulnar/radial deviation, elbow flexion/extension, and shoulder horizontal abduction/adduction). We asked participants to push with the corresponding arm segment (hand, forearm, or upper arm) against a fixed object that opposed motion around each degree of freedom of interest. The arm posture during the MVC task was similar to the baseline posture in the virtual polishing task.

In each session, before the virtual polishing task, participants were shown two videos. The first video showed the expert performing a real polishing operation. The second video showed an example participant performing a trial of the virtual polishing task. Both videos illustrated the polishing operation as a cyclic motion of the arms and the polished object from side to side while lightly contacting the grinder. Participants in the real feedback group were instructed to make the polished object as smooth as possible while also attempting to maximize the EMG score. Participants in the null feedback group were only instructed to make the polished object as smooth as possible. Additionally, participants in both groups were encouraged to explore different movement and force patterns to increase their scores. Participants in the real feedback group were informed that this exploration could influence the EMG score.

Each session of the virtual polishing task consisted of 60 trials, each with a duration of 30 s. Before each trial, participants moved each end-point of the manipulanda, indicated by white circles, to its initial position, indicated by red rings in the virtual environment. The initial position was centered on the display and close to the participant’s torso. The distance between the initial positions of each manipulandum end-point corresponded to the size of the polished object. At this stage, only the initial position indicators were visible, and no forces were generated by the manipulanda. After holding the manipulanda for 5 seconds at their initial positions, the polished object and the grinder became visible, and the simulated physics of the task were activated. The shape of the polished object was randomly generated at the beginning of each trial. The appearance of the virtual objects prompted the participants to begin the task. At the end of the trial the simulated physics were deactivated and the grinder disappeared. Participants could observe the resulting shape of the polished object and rest before starting the next trial. Participants were required to rest at least 5 seconds between trials, but rested 10.2 seconds (SD 4.0) on average.

### 2.8. Data analysis

#### 2.8.1. Synergy extraction

We used non-negative matrix factorization (NMF) (Lee and Seung, 1999) to extract muscle synergies from the EMG signals collected during the actual polishing task with the expert, and the virtual polishing task with the experiment participants. In our main analysis, the processed EMG signals from muscles of both arms were pooled together to extract bimanual synergies. In the case of the expert polisher, synergies were extracted for all number of synergies *N* from 1 to 16. We selected the number of synergies by finding the smallest *N*_b_ that resulted in a reconstruction quality R^2^ of the processed EMG of at least 0.90 (Berger et al., 2013; Barradas et al., 2020). We used the expert’s extracted synergies in the bimanual analysis to compute the EMG space similarity score during the experimental sessions. However, we decided to use a larger number of synergies *N*_EMG_ for the EMG score, as a pilot test showed that the EMG score was predominantly negative when *N*_b_ was used. We then extracted muscle synergies offline from each participant and experimental session. We set the number of synergies to *N*_EMG_ for each participant for comparison to the expert’s synergies.

We also conducted a secondary synergy analysis in which the processed EMG signals from the muscles of each arm were analyzed separately to extract unimanual synergies for each arm. The number of synergies *N*_u_ for each arm of the expert polisher was selected as described above, except that *N*_u_ was taken from 1 to 8. The number of synergies from each participant, experimental session, and arm were set to *N*_u_ for comparison to the expert’s synergies. Note that the unimanual synergies were not used to compute the EMG space similarity score.

#### 2.8.2. Muscle synergy similarity metric

We performed a similarity analysis between the expert’s muscle synergies and participants’ synergies extracted from each experimental session to assess changes in the synergies in both the bimanual and unimanual analyses. We defined the similarity between two different set of synergies as the average cosine of the angle between individual corresponding synergies in the two sets:


(12) $$s\,i\,m=\frac{1}{N}\,\sum_{i=1}^{N}\cos\theta_{i}=\frac{1}{N}\,\sum_{i=1}^{N}\frac{{\text{s}}_{i\,1}\cdot {\text{s}}_{i\,2}}{||{\text{s}}_{i\,1}||\,||{\text{s}}_{i\,2}||}$$

where *N* is the number of synergies in the set, θ is the angle between two individual synergies when considering them as a vector, and **s**_i1_ and **s**_i2_ are matched individual synergies to be compared represented as vectors. For each session, we matched individual synergies of the expert and the participants by computing the cosine similarity between each synergy in the expert’s synergy set and all other synergies in the participant’s synergy set. The pair of synergies with the largest similarity was considered a match, and was removed from the synergy sets. The process was then repeated with the remaining synergies in each set until all synergies in one set had been matched to a synergy in the other set. For each participant, we computed the muscle synergy similarity metric separately for each session in the experiment.

#### 2.8.3. Muscle synergy importance

We calculated the relative contribution of each extracted muscle synergy to the reconstruction of the EMG activity of the expert. Given that muscle synergies computed using NMF are not necessarily linearly independent, it is generally not possible to assess the contribution of each synergy by reconstructing the EMG using only the synergy of interest as in other matrix factorization algorithms like PCA. Instead, we defined the contribution of each synergy in terms of a synergy importance metric as


(13) I⁢M⁢Ps⁢i=1-R2s⁢r⁢iR2s⁢f

where IMP_si_ is the synergy importance of synergy *i* = 1, …, *N*, R^2^_sri_ is the reconstruction quality achieved with a reduced synergy set, from which synergy i has been removed, and R^2^_sf_ is the reconstruction quality achieved with the full synergy set. Therefore, larger values of the importance metric indicate a larger contribution of the synergy of interest to EMG reconstruction. To compute R^2^_sri_, we recalculated the synergy coefficients for the reduced synergy set that optimally reconstruct the EMG by performing a non-negative linear regression, as described in Equation 10.

We also applied this metric to participants in the virtual task using the expert’s muscle synergies. This allowed us to measure the degree to which the participants’ EMG can be explained by individual synergies of the expert. For each participant, we computed the muscle synergy importance metric separately for every session in the experiment.

#### 2.8.4. Polishing attack angle

The attack angle in a polishing operation is the angle formed between the grinder and the surface of the polished object. The variability of the attack angle is an indicator of the skill level in the polishing task (Kodama et al., 2015; Tsujiai et al., 2015). Therefore, we measured the attack angle that subjects used during the experiment. Given that the grinder in the virtual task is static and parallel to the horizontal in the task, the attack angle is determined by the orientation of the polished object with respect to the horizontal. For each participant, we calculated the standard deviation of the attack angle within each trial, and averaged the result of all trials within one session.

#### 2.8.5. Statistical analysis

The main outcome variables of the experiment were the average smoothness and EMG space similarity scores across learners in both the real and null feedback groups. Secondary outcome variables were the average variability of the polishing attack angle, the muscle synergy similarity and the muscle synergy importance. We assessed differences in the outcome variables using mixed two-factor ANOVA tests with trial session as the within-subjects factor (with five levels: sessions 1–5), and feedback type as the between-subjects factor. In cases where the sphericity assumption of the mixed ANOVA test was not met, we used the Greenhouse-Geisser correction. Furthermore, in cases where the outcome variables did not satisfy the normality assumptions of the ANOVA test, we used the non-parametric ANOVA-type statistic (ATS) implemented in the nparLD package in R (Noguchi et al., 2012). The significance threshold was set at *p* = 0.05. We performed *post-hoc* tests with Bonferroni corrections.

## 3. Results

### 3.1. One muscle synergy of the expert predominantly contributes to EMG reconstruction

**FIGURE 2:**
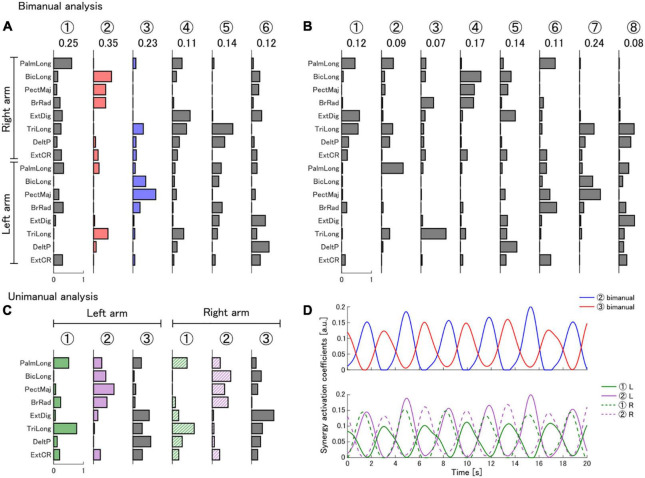
Muscle synergies extracted from the expert during a real polishing operation. **(A)** Bimanual muscle synergies extracted according to the synergy selection criteria (*R*^2^ > 0.90). The number of synergies was determined as *N*_b_ = 6. Synergies were extracted from the palmaris longus (PalmLong), biceps brachii long head (BicLong), pectoralis major (PectMaj), brachioradialis (BrRad), extensor digitorum (ExtDig), triceps brachii long head (TriLong), deltoid posterior (DeltP), and extensor carpi radialis (ExtCR) muscles of both arms. **(B)** Bimanual muscle synergies used to compute the EMG similarity score in the virtual polishing task. We selected *N*_EMG_ = 8 to facilitate improvement in the task. Encircled numbers are labels indicating the synergy number. The value of the synergy importance metric within a synergy set is indicated below each synergy label. **(C)** Unimanual muscle synergies extracted separately from the left and right arms according to the synergy selection criteria. **(D)** Muscle synergy activation coefficients of a subset of synergies of the expert during a sample portion of a trial in the real polishing task. The upper and lower panels show the activations of bimanual and unimanual synergies, respectively. The color of the traces indicates the corresponding bimanual or unimanual synergy.

We analyzed the muscle activations of 8 muscles from both arms of an expert performing a real polishing operation. In the bimanual analysis, we identified 6 muscle synergies (*N*_b_ = 6), which achieved a reconstruction quality (R^2^) of 0.90 of the original EMG signals. We also quantified the contribution of each extracted synergy to the reconstruction quality of the expert’s EMG signals by calculating the synergy importance metric in the bimanual analysis. We found that synergy 2 played a predominant role in the reconstruction of the EMG signals (Figure 2A). Synergy 2 had an importance value of 0.35. which was 1.4 times larger than the synergy with the next highest importance (0.25).

### 3.2. Bimanual muscle synergies capture temporal coordination of unimanual synergies in the expert

In the unimanual analysis, we identified 3 muscle synergies in each arm (*N*_u_ = 3, R^2^_left_ = 0.91, R^2^_right_ = 0.90) (Figure 2C). The synergies in both arms had a largely equivalent composition, which we quantified as the cosine similarity between corresponding synergy pairs in both arms (syn 1: 0.95, syn 2: 0.93, syn 3: 0.87). A comparison between the bimanual and unimanual synergies revealed that unimanual left synergy 1 and unimanual right synergy 2 can be combined to form a bimanual synergy that highly resembles the important bimanual synergy 2 (cosine similarity: 0.92). Similarly, unimanual left synergy 2 and unimanual right synergy 1 can be combined to form a synergy that resembles bimanual synergy 3 (cosine similarity: 0.86). The activation coefficients of bimanual synergies 2 and 3 show that, during the task, the synergies are activated in an alternating pattern (Figure 2D). Furthermore, the activations of the unimanual synergies contained in bimanual synergies 2 and 3 are locked in phase with each other, and with their corresponding bimanual synergy (Figure 2D).

### 3.3. Real feedback group showed improvement in smoothness and EMG space similarity scores

**FIGURE 3:**
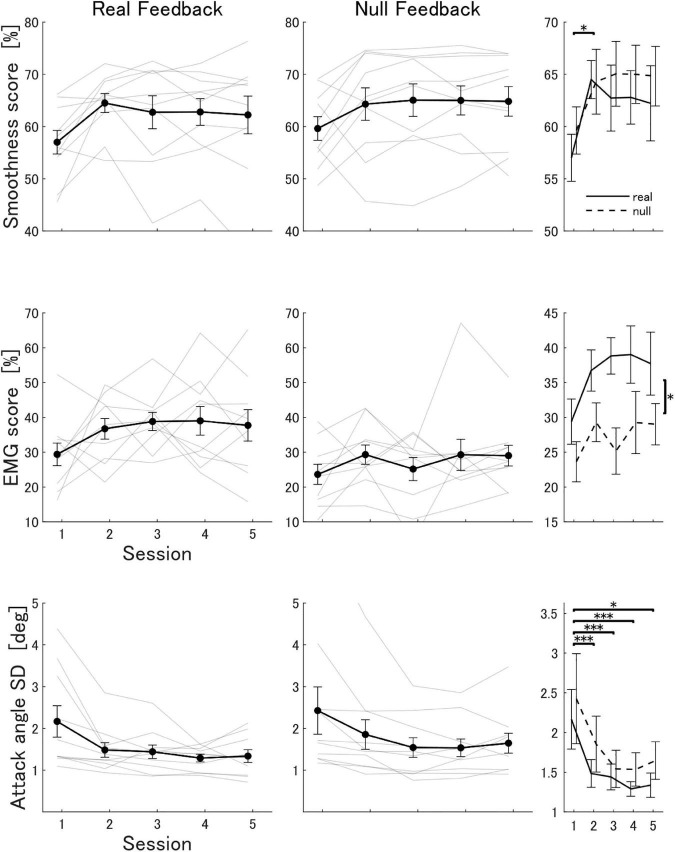
Progression of the smoothness score, EMG similarity score and attack angle variability in the real and null feedback groups. The attack angle variability was measured as the standard deviation of the attack angle across all trials in one session. Thick lines indicate the mean score across participants in each group. Light lines indicate scores for each participant. Error bars indicate the standard error. The third column summarizes statistical comparisons. Asterisks indicate significant differences between metrics: ^***^p < 0.001, *p < 0.05.

Participants in the real feedback group showed an improvement in the mean smoothness score throughout the experiment [session 1: 57.0 % (SD 7.1), session 5: 62.2 % (SD 11.4)]. Participants in the null feedback group also showed an improvement in the mean smoothness score [session 1: 59.6 % (SD 7.1), session 5: 64.8 % (SD 9.0)]. A mixed two-factor ANOVA analysis showed that there was no statistically significant interaction between the effects of training session and feedback type (real or null) on the mean smoothness score [*F*(2.14,38.55) = 0.323, *p* = 0.74]. The ANOVA analysis also determined that there was a statistically significant main effect of training session [*F*(2.14,38.55) = 5.79, *p* = 0.005], but not of feedback type [*F*(1,18) = 0.30, *p* = 0.59]. A *post-hoc* analysis confirmed a statistically significant difference in the mean smoothness score between training sessions 1 and 2 [paired *t*-test with Bonferroni corrections, *p* = 0.039]. Figure 3 shows the progression in the smoothness scores for all participants.

We used the extracted bimanual synergies from the expert with *N*_EMG_ = 8 to reconstruct the EMG signals of participants in the virtual polishing task and compute the EMG space similarity score (Figure 2B). The reconstruction quality R^2^ of the expert’s EMG by the synergy set with *N*_EMG_ = 8 was 0.94. Participants in the real feedback group showed an improvement in the mean EMG score throughout the experiment [session 1: 29.4% (SD 10.3), session 5: 37.7 % (SD 14.3)]. Participants in the null feedback group showed a smaller improvement in the mean EMG score [session 1: 23.6% (SD 9.1), session 5: 29.0% (SD 9.4)]. Due to the presence of outliers and non-normality in the data we used a non-parametric test (ATS). The ATS analysis (nparLD) showed that there was no statistically significant interaction between the effects of training session and feedback type on the mean EMG score [*F*(3.06, ∞) = 0.35, *p* = 0.79]. The ATS analysis also determined that there was a statistically significant main effect of feedback type [*F*(1.00, ∞) = 9.70, *p* = 0.002], but not of training session [*F*(3.06, ∞) = 2.05, *p* = 0.10]. Figure 3 shows the progression in the EMG score for all participants.

We also measured the variability in the attack angle throughout the experiment as the standard deviation of the attack angle across all trials in a session. Participants in the real feedback group showed a decrease in the mean variability of the attack angle [session 1: 2.2° (SD 1.2), session 5: 1.3° (SD 0.5)]. Similarly, participants in the null feedback group showed a significant decrease in the mean variability of the attack angle [session 1: 2.4° (SD 1.8), session 5: 1.6° (SD 0.7)]. An ATS analysis showed that there was no statistically significant interaction between the effects of training session and feedback type on the variability of the attack angle [*F*(2.01, ∞) = 0.31, *p* = 0.56]. The ATS analysis also determined that there was a statistically significant main effect of training session [*F*(2.01, ∞) = 6.22, *p* = 0.002], but not of feedback type [*F*(1.00, ∞) = 0.31, *p* = 0.58]. A *post-hoc* analysis confirmed a statistically significant difference in the mean variability of the attack angle between training sessions 1 and 2 [ATS with Bonferroni corrections, *p* < 1 × 10^–9^], 1 and 3 [*p* < 1 × 10^–4^], 1 and 4 [*p* < 1 × 10^–4^], and 1 and 5 [*p* = 0.04], but no difference between the rest of the session pairs. Figure 3 shows the progression in the variability of the attack angle for all participants. Interestingly, the variance across subjects of the standard deviation of the attack angle, that is, a measure of the variability across subjects of the variability of the attack angle within subjects, was significantly smaller in the real feedback group than in the null feedback group during session 4 (*F*-test for equality of variances, *p* = 0.021).

### 3.4. Real feedback group showed increased importance and similarity to one muscle synergy of the expert

**FIGURE 4:**
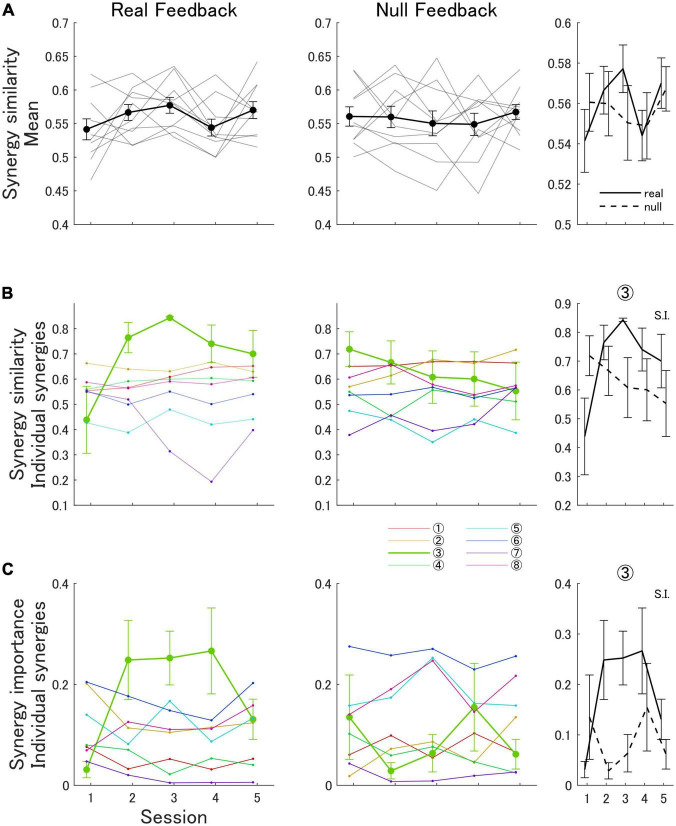
Bimanual synergy similarity and importance metrics in the real and null feedback groups. **(A)** Mean synergy similarity metric. Thick lines indicate the mean score across participants in each group. Light lines indicate scores for each participant. Error bars indicate the standard error. **(B)** Cosine similarity metric of individual synergies (1–8). **(C)** Synergy importance of individual expert synergies (IMP_s1_–IMP_s8_). The third column summarizes statistical comparisons. The S.I. legend indicates a significant interaction between session number and feedback type on the outcome variable.

The muscle synergies of participants in the real feedback group did not appreciably become more similar to the expert’s bimanual synergies (*N*_EMG_ = 8) as a whole throughout the experiment according to the mean muscle synergy similarity metric [session 1: 0.54 (SD 0.05), session 5: 0.57 (SD 0.04)]. This was also true for participants in the null feedback group [session 1: 0.56 (SD 0.05), session 5: 0.57 (SD 0.03)]. A two-way ANOVA analysis showed that there was no statistically significant interaction between the effects of training session and feedback type on the mean synergy similarity to the expert [*F*(4,72) = 0.93, *p* = 0.45]. Additionally, the analysis determined no statistically significant main effects of training session [*F*(4,72) = 1.16, *p* = 0.34] or feedback type [*F*(1,18) = 0.04, *p* = 0.85]. Figure 4A shows the progression of the synergy similarity metric of participants in the real feedback and null feedback groups. Supplementary Figure 1 shows the synergies extracted from a representative participant in the real feedback group in sessions 1 and 5.

We also examined the cosine similarity of participant’s individual muscle synergies to the corresponding expert synergy (*N*_EMG_ = 8) during each session of the experiment. In the bimanual analysis, we found that only synergy 3 showed a trend for increasing similarity in the real feedback group (Figure 4B). Participants in the real feedback group showed an increase in the cosine similarity of synergy 3 [session 1: 0.44 (SD 0.42), session 5: 0.70 (SD 0.29)], whereas participants in the null feedback group showed a decrease [session 1: 0.72 (SD 0.22), session 5: 0.55 (SD 0.36)]. An ATS analysis showed that there was a statistically significant interaction between the effects of training session and feedback type on the similarity of synergy 3 [*F*(3.35, ∞) = 2.71, *p* = 0.037]. A statistically significant main effect of feedback type was found [*F*(1.00, ∞) = 4.02, *p* = 0.045], but not of training session [*F*(3.35, ∞) = 0.89, *p* = 0.45].

**FIGURE 5:**
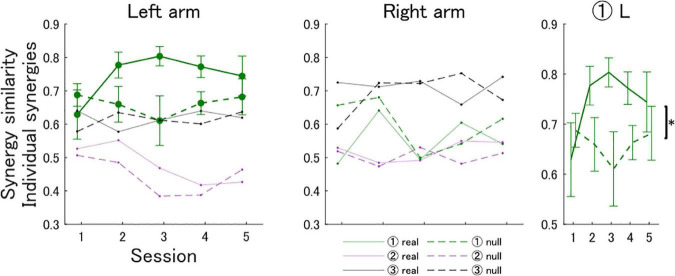
Cosine similarity metric of individual unimanual synergies in the real and null feedback groups. The first and second panels show the similarity metric for the left and right arms, respectively. Solid and dashed lines show the results for the real and null feedback groups, respectively. The third column summarizes statistical comparisons. The asterisk indicates a significant effect of feedback type on the similarity of synergy 1 of the left arm to the corresponding expert synergy.

Similarly, in the unimanual analysis we found that only synergy 1 of the left arm showed a trend for increasing similarity in the real feedback group (Figure 5). Participants in the real feedback group showed an increase in the cosine similarity of synergy 1 [session 1: 0.63 (SD 0.23), session 5: 0.74 (SD 0.19)], whereas participants in the null feedback group showed no increase [session 1: 0.69 (SD 0.11), session 5: 0.68 (SD 0.17)]. An ATS analysis showed that there was no statistically significant interaction between the effects of training session and feedback type on the similarity of synergy 1 [*F*(3.74, ∞) = 1.08, *p* = 0.36]. A statistically significant main effect of feedback type was found [*F*(1.00, ∞) = 5.06, *p* = 0.025], but not of training session [*F*(3.74, ∞) = 0.68, *p* = 0.59].

Additionally, we examined the importance of the expert’s bimanual synergies in reconstructing the EMG of the participants in the virtual polishing task. In particular, we examined the importance of synergy 3 (IMP_s3_), as it was the only synergy for which similarity increased throughout the experiment (Figure 4C). We found that IMP_s3_ increased for participants in the real feedback group [session 1: 0.03 (SD 0.05), session 5: 0.13 (SD 0.12)]. In contrast, IMP_s3_ did not increase for participants in the null feedback group [session 1: 0.14 (SD 0.26), session 5: 0.06 (SD 0.1)]. An ATS analysis showed that there was a statistically significant interaction between the effects of training session and feedback type on IMP_s3_ [*F*(3.37, ∞) = 4.64, *p* = 0.002]. Statistically significant main effects of feedback type [*F*(1.00, ∞) = 4.41, *p* = 0.036] and training session [*F*(3.37, ∞) = 2.84, *p* = 0.031] were found.

Interestingly, out of all synergies in the expert synergy set with *N*_b_ = 6, synergy 3 of the set with *N*_EMG_ = 8 is most similar to synergy 2, the predominant synergy (cosine similarities between synergy 3 from set *N*_EMG_ = 8 and synergies from set *N*_b_ = 6: syn 1: 0.37, syn 2: 0.71, syn 3: 0.15, syn 4: 0.60, syn 5: 0.30, syn 6: 0.35).

## 4. Discussion

Learning a new motor skill entails concurrently acquiring kinematic and kinetic movement patterns to satisfy the goals of a task. Augmented feedback that contains kinematic and/or kinetic information provided by a coach or an augmented reality system can facilitate motor skill learning (Young and Schmidt, 1992; Vander Linden et al., 1993; Lauber and Keller, 2014). Humans are able to simultaneously meet kinematic and kinetic task goals by appropriately modulating muscle activations, which control the stiffness of the limbs during a movement, and set the level of compliance in interactions with tools and other objects (Hogan, 1985b). Therefore, EMG from the muscles involved in the task can provide information about the kinematics and kinetics of the task. Here, we propose the EMG space similarity feedback, a new kind of augmented feedback that compares the EMG of learners to the EMG of an expert in the task. This kind of feedback could provide implicit comparisons between the kinematics and kinetics of movements of learners and the expert. The EMG space similarity feedback could be especially relevant for skills that have an important kinetic component, which in contrast to the kinematic component, is hard to evaluate by external observers without the use of special equipment.

We tested the EMG space similarity feedback in a virtual environment that simulates a polishing operation with a grinder, which requires precise kinematic and kinetic patterns of movement (Kodama et al., 2015). We divided participants into two groups: real and null feedback groups. The real feedback group was provided with the EMG space similarity feedback at all times, whereas the null feedback group was not. While both groups were able to improve the EMG score throughout the experiment, the real feedback group consistently showed larger EMG scores. However, we found that the groups did not differ significantly in their performance in the task, as measured by the smoothness score (Figure 3). This suggests that the smoothness objective of the virtual polishing task may be achievable independently of the way muscles are engaged in the task. Therefore, a variety of muscle activation strategies may be able to produce equivalent smoothness results in the virtual task. In any case, we have demonstrated that the EMG space similarity score can induce the use of muscle activation patterns that are increasingly consistent with a template synergy set.

Theories of motor skill learning posit that the acquisition of a skill is the result of the adjustment of a variety of processes and computations in the CNS (Willingham, 1998). Under this view, strategic and perceptual-motor integration processes must first transform the explicit goal of smoothening the polished object into lower level goals that correspond to suitable kinetics and kinematics for the task. Therefore, optimization of these processes may not directly cause improvements in task performance, especially in the early stages of learning, as other processes (i.e., sequential and/or dynamic processes) may need to be further tuned to exploit the output of the strategic and integration processes. The EMG space similarity feedback could contribute to these complex processes, as it can guide learners to generate muscle activations in a desired space, which can be associated to a corresponding kinetic and kinematic space that includes the goal actions. Thus, the EMG score may contribute to the overall process of motor skill learning.

We expected that the EMG score would reflect the similarity between the expert’s and the learner’s muscle synergies. Indeed, the EMG space similarity score is a local approximation to the global variance accounted for (VAF) metric, which has been described in previous research as a holistic measure of the similarity of two synergy sets (Perreault et al., 2008; Roh et al., 2013). The only difference is that our EMG score continuously evaluates the quality of reconstruction of EMG within short time windows, as opposed to using the whole data set at once. However, we did not find any improvements in the muscle synergy similarity metric that mirrored the improvements in the EMG score by the real feedback group. This underscores that both metrics quantify synergy similarity in different ways. Namely, two different synergy sets may produce muscle activations in increasingly overlapping regions of the muscle activation space without necessarily becoming more similar themselves. One way to visualize this is that a vector space may have multiple bases, and the bases can be orthogonal to each other. An additional reason is that the global VAF metric considers the variance of the data corresponding to each synergy to determine the similarity between synergy sets, whereas the muscle synergy similarity metric does not (Perreault et al., 2008). Therefore, the effect of individual synergies that explain larger portions of the variance is prioritized in the global VAF metric, as discussed below. These observations suggest that the EMG score cannot promote the directed learning of muscle synergies strictly, but instead allows to approximate the desired muscle activation space.

Illustrating the preference of the EMG score for important individual synergies, we found that participants in the real feedback group showed a tendency to improve the similarity of a single synergy to one of the expert’s synergies (synergy 3 from the extended set with *N*_EMG_ = 8) (Figure 4B and Supplementary Figure 1). Moreover, we also found that synergy 3 of the expert became increasingly important (Equation 13) in reconstructing the EMG signals of participants in the real feedback group throughout the experiment (Figure 4C). However, we did not observe this effect in the null feedback group. Therefore, the real feedback group acquired an expert-like synergy during training.

We can further explain the nature of the synergy similarity and EMG space similarity metrics by considering the rest of the individual synergies. While one synergy increased in similarity to synergy 3 of the expert, another synergy decreased in similarity to synergy 7, and the rest of the synergies did not show appreciable changes (Figure 4). Therefore, any increase in the mean synergy similarity score was canceled. However, the importance of synergy 7 was very low throughout the experiment, suggesting that the observed increases in the EMG score reflected the acquisition of synergy 3 of the expert.

Interestingly, synergy 3 from the expert’s set with *N*_EMG_ = 8 highly resembles synergy 2 from the set with *N*_b_ = 6, which is the synergy with the highest importance in the *N*_b_ = 6 set (Figure 2). In fact, synergy 2 in the set with *N*_b_ = 6 appears to have fragmented into synergies 3 and 4 in the set with *N*_b_ = 8, which is common when increasing *N* in the non-negative matrix factorization algorithm (Cheung et al., 2012; Barradas et al., 2020). This explains why the importance of synergies 3 and 4 in the set with *N*_b_ = 8 is reduced with respect to their parent synergy in the set with *N*_b_ = 6. That is, in our synergy importance metric, removing one of the fragmented synergies while leaving the other one in the set allows a better reconstruction of the EMG activity in the task than if both fragments were removed, reducing the importance metric of both fragments. However, synergy 3 (and not synergy 4) in the *N*_EMG_ = 8 set encodes a strong bimanual coordination pattern that is also characteristic of synergy 2 in the set with *N*_b_ = 6, and which may be essential to explain its high importance. Therefore, participants in the real feedback group not only acquired an expert-like synergy, but they acquired the most important synergy within the expert’s synergy set.

It is possible that the expert-like synergy that participants in the real feedback group acquired is associated with kinematic and/or kinetic patterns that are relevant to both the actual and the virtual tasks. This synergy is composed mainly of the co-activation of the left triceps long head, an elbow extensor, and the right brachioradialis, an elbow flexor. Therefore, this synergy could be involved in producing a counter-clockwise torque around the center of the polished object when held bimanually. The grinder also produces torques around the object during contact with off-center spots on the object. Therefore, this synergy could be important to counter the torque from the grinder, stabilizing the object during contact. This is reasonable, as an important symmetric synergy that could stabilize the object in the opposite direction is present in the expert’s synergy set with *N*_b_ = 6 (synergy 3). Synergy 7 in the synergy set with *N*_EMG_ = 8 (or synergy 3 in *N*_b_ = 6) is symmetric to synergy 2 in the set with *N*_b_ = 6, suggesting that the expert uses symmetric patterns to stabilize the object. However, subjects in the real feedback group did not show muscle activation patterns consistent with this synergy. This again highlights the fact that the EMG space similarity feedback does not seem to be able to promote learning desired muscle synergies directly.

Here, we computed the EMG space similarity feedback *via* a bilateral muscle synergy analysis that pools signals from both arms (Jarrassé et al., 2014; Botzheim et al., 2021). This analysis can capture timing relationships between synergies of both arms as a spatial structure, which enables the EMG score to provide information about the coordination of both arms (Figure 2). However, a large body of evidence indicates that limb-specific synergies are encoded in spinal circuits across species (Kargo and Giszter, 2008; Kargo et al., 2010; Giszter, 2015; Takei et al., 2017; Yang et al., 2019; Cheung and Seki, 2021). An EMG score based on a unimanual synergy analysis would not be able to convey the necessary information about bimanual coordination to effectively learn expert-like muscle activation patterns. However, a unimanual synergy analysis of both arms revealed that subjects in the real feedback group showed increased similarity to one of the left arm synergies of the expert. Interestingly, this learned unimanual synergy constituted the left arm component of the important bimanual synergy discussed above (synergy 2 in the set with *N*_b_ = 6, and synergy 3 in the set with *N*_EMG_ = 8). Therefore, the unimanual analysis allowed us to observe that, in addition to an increased interarm coordination, the acquisition of the important bimanual synergy was due to the acquisition of a left arm synergy, but not a right arm synergy (Figure 5). This warrants further research, as the EMG space similarity feedback may have an effect on arm specialization *via* interarm differences in synergy control (Sainburg, 2005).

The attack angle between the grinder and the polished object has been previously identified as a kinematic variable that reflects expertise in a polishing task (Kodama et al., 2015). Expert polishers show smaller variability in the attack angle than inexperienced people. Because of the interaction forces between the grinder and the polished object, reducing variability in the attack angle most likely also involves precise kinetic patterns. We found that participants in both the real and null feedback groups showed a reduction in the variability of the attack angle throughout the experiment. It is notable that participants tended to modify their kinematic patterns in a way that characterizes expertise in the task without receiving explicit instructions to do so. Interestingly, the real feedback group appeared to be more consistent in reducing the variability in the attack angle during the experiment, as the variance among subjects in this metric was smaller than for the null feedback group. This may indicate increased stability in the motion against the grinder.

Training paradigms to shape muscle activations using EMG as both a control and an augmented feedback signal have been proposed previously. Feedback that promotes changing the timing of peak EMG of a single muscle during a cycling task has been shown to also promote time shifts in the activations of other muscles, resulting in novel muscle activation patterns (Torricelli et al., 2020). However, it is unclear whether these novel muscle activations can be shaped to a desired pattern, or bring about useful or even new kinematic or kinetic movement patterns. In a different kind of tasks, the position of objects in a virtual environment can be controlled as a function of EMG. The function can be defined so that successful completion of the task requires decorrelating the activity of two or more muscles (Wright et al., 2014; Mugler et al., 2019), or learning entirely new muscle activations patterns (Berger et al., 2021). Therefore, whereas learning in these tasks may reshape muscle activation patterns, it is unclear whether the newly learned patterns are transferable to more ecological tasks. In contrast, the EMG space similarity feedback can be used during actual ecological tasks, where it complements task performance feedback. Therefore, as long as the task constraints are reasonably met, it can assist in reshaping muscle activations in a task-relevant way.

One limitation of our study is that there are different task constraints in the actual and virtual polishing environments. Namely, in the actual polishing operation, the expert sat in front of the grinder, which was placed at the height of the hips in a sitting posture. This allowed him to perform the polishing operation while supporting his forearms on top of his upper legs. Consequently, the side-to-side polishing motion involves not only arm, but also leg movements. Additionally, contact between the grinder and the polished object produces friction forces in the vertical direction. In contrast, in the virtual polishing environment, the polishing movement was restricted to a horizontal plane located at approximately the height of the sternum when sitting upright. Therefore, in the virtual task the weight of the arms is not supported and the legs were not involved in the task. Furthermore, our robotic system only produced forces on the horizontal plane. Differences in the task biomechanics may entail differences in the optimal arm kinematics and kinetics in the actual and virtual tasks. Moreover, differences between the sizes of body parts of the expert and the learners may also entail different biomechanical constraints even within the same task, real or virtual. This suggests that the EMG space similarity feedback may contain information about the actual task that is inappropriate or irrelevant for the virtual task. However, even if the expert or template synergies corresponded to an arbitrary task unrelated to the task of interest, our results show that learners are able to use the EMG space similarity feedback to increasingly move their muscle activation patterns into the space spanned by the expert synergies.

This could offer an explanation to the observation that some participants in the real feedback group showed a decline in the smoothness score after reaching a peak, even though they were able to keep improving the EMG score. Because of the different biomechanical contexts of the actual and virtual tasks, attempting to further imitate the expert’s EMG patterns could be difficult and even detrimental to performance. However, the positive results up to session 3, and the lack of evident differences in performance between the real and null feedback groups suggest that the EMG score conveys sufficiently compatible information between both tasks. This may involve force application patterns onto the grinder that are universally useful in polishing-like operations.

The emphasis on important synergies and the possible presence of inappropriate synergies for the virtual task in the expert’s synergy set suggest an improvement to the implementation of the EMG space similarity score. Reducing the expert synergy set to appropriate and/or important synergies would produce a score that is more related to useful kinetic and kinematic movement patterns. However, it must be taken into account that reduced synergy sets cannot account for some important task-space components in some tasks (Barradas et al., 2020). Another improvement to the EMG score would be to include information about the timing of the expert synergies during the task. This would provide a more direct way to indicate the desired kinetic and kinematic patterns. However, this can only work under the assumption that the expert patterns of muscle activation have been previously acquired. Therefore, we envision the current EMG space similarity score as a way to promote the acquisition of expert-like muscle activation patterns, that is, the building blocks of movement. Upon the acquisition of these building blocks, further training on how to use these blocks is needed.

In the future we plan to study the transferability of the polishing skill within the virtual environment. Namely, it is necessary to verify whether subjects in the real feedback group are able to retain the improved performance levels after the removal of the EMG space similarity feedback in the virtual task. Next, we also plan to study the transferability of the polishing skill between the actual and virtual environments. In a first stage, the expert polisher would perform the task in the simulated environment. Such a study would allow us to directly measure the kinematic and kinetic patterns that the expert uses during the task and compare them to the patterns acquired by the learners. This would confirm whether the movement patterns of the expert are actually conveyed to the learners through the EMG space similarity feedback. In a second stage, the learners would perform a baseline session in the real environment followed by training in the virtual environment, and finally, a transfer test in the real environment. Improvements in the transfer test would not only further confirm that the EMG score teaches appropriate kinematic and kinetic patterns, but would also establish a basis for augmented reality training of highly skilled technicians.

Overall, our results suggest that augmented feedback in the form of an EMG space similarity score can facilitate the acquisition of expert-like muscle activation patterns. Thus, the EMG space similarity score may assist in learning a complex skill by placing the muscle activations of learners in a space that could facilitate producing expert-like motions. Thus, the EMG space similarity feedback could be a useful tool to facilitate motor training for technicians and athletes. Further research is necessary to determine its applicability in a broader range of tasks, and to investigate the mechanisms through which it operates. A promising feature of the EMG score is that it probably conveys feedback about desired task kinetics, which usually requires especially instrumented equipment. EMG measurement systems can be used in a wider range of contexts than such equipment. Therefore, it is possible that the EMG score could be used as a substitute or in parallel to more specialized equipment to provide kinetic feedback.

## Data availability statement

The raw data supporting the conclusions of this article will be made available by the authors, without undue reservation.

## Ethics statement

The studies involving human participants were reviewed and approved by the Tokyo Institute of Technology Human Subjects Research Ethics Review Committee. The patients/participants provided their written informed consent to participate in this study.

## Author contributions

VB and YK conceived and designed the research, edited and revised the manuscript, and interpreted the results of experiments. VB and WC performed the experiments. VB analyzed the data and drafted the manuscript. All authors approved the final version of the manuscript.
