# Supplementary material for: EMG space similarity feedback promotes learning of expert-like muscle activation patterns in a complex motor skill
Source: Front Hum Neurosci. 2023 Jan 20;16:805867. doi: 10.3389/fnhum.2022.805867 (PMC9897456; doi:10.3389/fnhum.2022.805867)
Supplement: Supplementary file 1 [file Data_Sheet_1.DOCX]

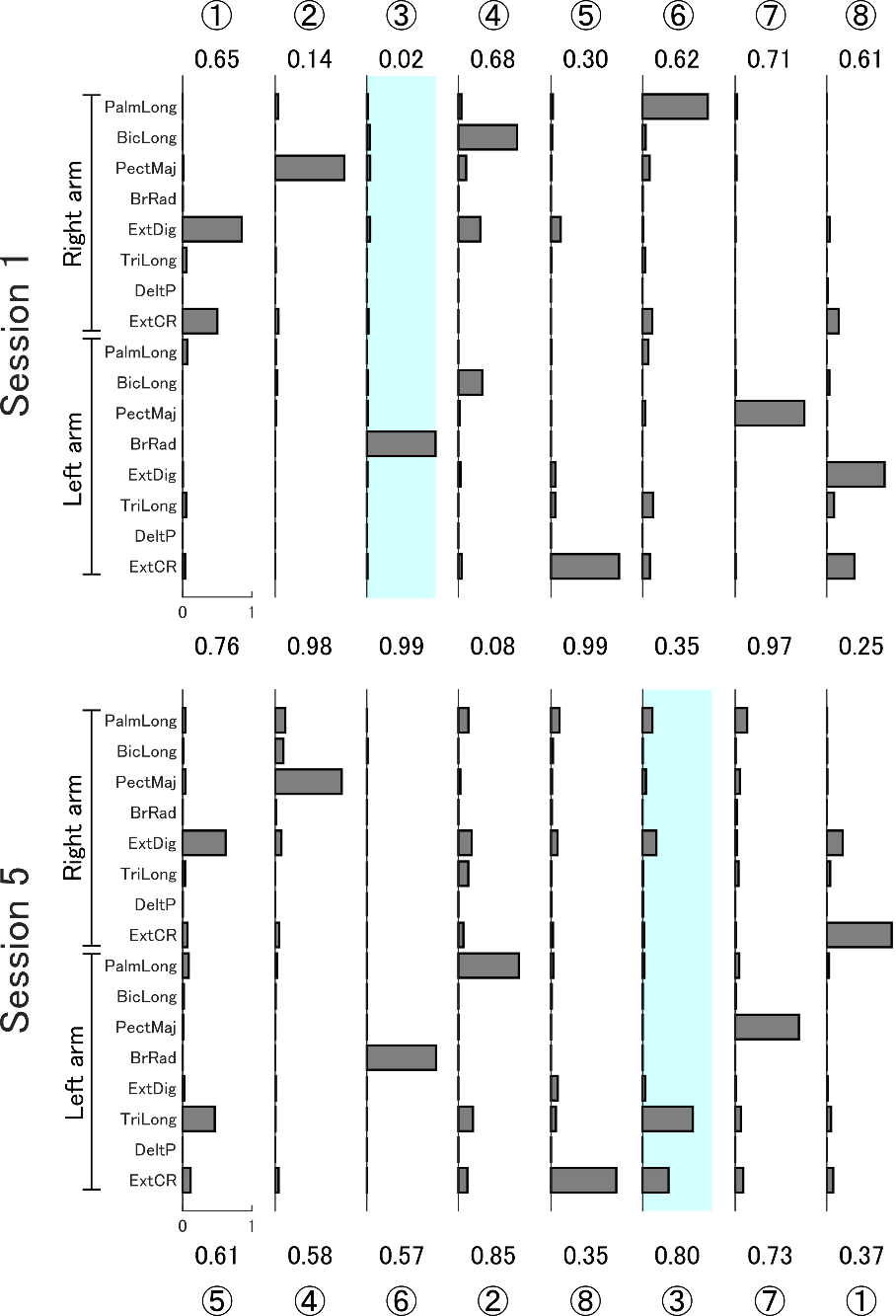


Figure S1. Muscle synergies extracted from a representative participant in the real feedback group in sessions 1 and 5 of the virtual polishing task. Synergies in session 5 are arranged so that they match a similar synergy in session 1 according to the cosine similarity metric. The similarity between each pair of matched synergies in sessions 1 and 5 is indicated in the center of the figure. Encircled numbers indicate the expert synergy in the set with N*_EMG_* = 8 to which each individual synergy was matched. The similarity to the corresponding expert synergy is indicated above or below the encircled number. Notice that although some synergies in sessions 1 and 5 are similar, they are matched to a different expert synergy. This is because these synergies do not closely resemble an expert synergy, so they may be matched to different expert synergies depending on the matches between other synergies. Additionally, notice that the synergy matched to synergy 3 of the expert in session 1 is not similar to the expert synergy (cosine similarity: 0.02), whereas in session 5 the similarity has increased dramatically (cosine similarity: 0.80). Therefore, the synergies in sessions 1 and 5 that were matched to expert synergy 3 (highlighted synergies in each set) do not resemble each other. This suggests that the synergy that corresponds to synergy 3 of the expert in session 5 did not exist in session 1 and was acquired during training.
